# Effects of Light Spectral Quality on the Micropropagated Raspberry Plants during Ex Vitro Adaptation

**DOI:** 10.3390/plants10102071

**Published:** 2021-09-30

**Authors:** Ivan G. Tarakanov, Anatoly A. Kosobryukhov, Daria A. Tovstyko, Alexander A. Anisimov, Alla A. Shulgina, Nikolay N. Sleptsov, Elena A. Kalashnikova, Andon V. Vassilev, Rima N. Kirakosyan

**Affiliations:** 1 Department of Plant Physiology, Russian State Agrarian University—Moscow Timiryazev Agricultural Academy, Timiryazevskaya Str., 49, 127550 Moscow, Russia; tov.dasha@mail.ru (D.A.T.); alanis152@mail.ru (A.A.A.); inkss@mail.ru (N.N.S.); 2 Institute of Basic Biological Problems, Russian Academy of Sciences, Institutskaya Str., 2, 142290 Pushchino, Russia; kosobr@rambler.ru; 3 Department of Biotechnology, Russian State Agrarian University—Moscow Timiryazev Agricultural Academy, Timiryazevskaya, 49, 127550 Moscow, Russia; alja.shulgina@yandex.ru (A.A.S.); kalash0407@mail.ru (E.A.K.); mia41291@mail.ru (R.N.K.); 4 Department of Plant Physiology, Biochemistry and Genetics, Faculty of Agronomy, Agricultural University, Mendeleev Str., 12, 4000 Plovdiv, Bulgaria; andon.vasilev@abv.bg

**Keywords:** raspberry, clonal micropropagation, ex vitro, adaptation, light spectral quality, photosynthesis, photomorphogenesis, far-red light, green light, light-emitting diodes

## Abstract

This work focuses on developing light environments for the effective regulation of morphogenesis and ex vitro conditions adaptation in micropropagated raspberry plants on the basis of photomorphogenetic control of physiological processes using light-emitting diodes (LEDs). In experiments with cloned plants growing ex vitro in stressful conditions during acclimation, the effects of optical radiation of various spectral combinations from different photosynthetically active radiation (PAR) spectral regions were studied. The data on the plant development and state of the photosynthetic apparatus, features of photosynthetic gas exchange and transpiration, accumulation of photosynthetic pigments, light curves of photosynthesis, and data on growth processes in light modes using combined quasimonochromatic radiation (either mixture of red, green, and blue light or red, far-red, and blue light) with various ratio of the distinct spectral regions were obtained. Photosynthetic apparatus functional activity under different light conditions was studied with chlorophyll fluorescence determination, and plant stress responses to growing under artificial spectral light conditions were characterized. The experiments were accompanied by detailed plant phenotyping at the structural and functional levels. Plant acclimation and photosynthetic improvements in response to added far-red and green light wavelengths to the main red-blue spectrum have been elucidated.

## 1. Introduction

Our photobiological studies are aimed at developing effective methods for regulating morphogenesis and obtaining high-quality planting material of woody garden plants propagated vegetatively using in vitro technologies, based on photomorphogenetic regulation of physiological processes using light-emitting diodes (LEDs). The use of blue and red LEDs with spectral power distributions optimal for photosynthesis affords considerable energy savings. On the other hand, the recent availability of high-brightness diodes emitting light of other spectral regions opens new perspectives for precise horticultural lighting, including plant clonal micropropagation in vitro and ex vitro. The studies on the physiological mechanisms of light action in plants, taking into account the new experimental possibilities opened with the use of LEDs, allow us to approach the development of light modes at a fundamentally new level [1], ensuring the success of plant adaptation in vitro, as well as their ability to overcome the stress of transfer to ex vitro conditions in a controlled light environment. These are different plant model systems with various levels of integrity: ex-plant in vitro and intact whole plant ex vitro.

Light spectral composition role in the control of plant morphogenesis (including in vitro growth) is well recognized. Changes in light spectral environment influence plant shoot and root formation, growth rate, and metabolic processes direction [2,3]. Various spectral regions of PAR also have differing effects on photosynthesis on a quantum yield basis [4,5,6,7,8]. Red and blue are generally recognized as the most important light regions necessary for plant development and growth [9]. It was also observed that red and blue wavelengths are more favorable for photosynthesis than green wavelengths. Treatments with quasimonochromatic light, red or blue, have shown that plants raised in red light are usually characterized by relatively poor growth and low photosynthetic capacity, unresponsive stomatal conductance, low specific leaf weight (leaf thickness), and low maximum quantum efficiency of photosystem II [10,11,12,13]. Blue light controls stomatal opening and transpiration, as well as preventing “red light syndrome” [14]. Blue light effects on the shoot architecture are usually resulting in a compact plant habit [15]. Blue light addition to red can improve plant photosynthetic effectiveness [13]. Furthermore, a combination of red and blue light in certain cases can result in synergetic effects in biomass accumulation [16]. Green LED light regulates leaf expansion, stem stretching, and stomatal conductance. Moreover, it has been shown that green LED light leads to growth stimulation and greater dry mass accumulation [17].

Besides light effects on plant photosynthetic responses, light spectral quality determines numerous photomorphogenetic responses in plants, providing better plant adaptation to the light environment of coenosis, seasonal changes, etc. First of all, plant photomorphogenesis depends on the ratio of blue, red, and far-red photon fluxes. At the same time, green light addition often enhances created artificial light environment effects on the morphophysiological processes in plants both in vitro and ex vitro [18,19].

Numerous studies have focused on the role of red, blue, and their combinations in the control of plant growth and development [11,20,21,22,23]. Relatively little research has been done on green light effects [16,24,25,26]. However, plant physiological responses can be the result of interactions between various light wavelengths; therefore, it is important to include green light in spectral quality studies [27]. Thus, it was found that green light can modulate blue light-induced phototropism [28]. Additionally, green light reversed blue light-induced stomatal opening [29,30]. Green light penetration and distribution in the plant canopy and inside the leaf due to its high transmittance and reflectance also deserves special attention [31]. Recently, studies have suggested further photosynthetic improvements by adding far-red (FR) wavelengths to red (R) spectra; for example, increasing FR light ratio promoted the growth of seedlings by increasing leaf expansion and whole-plant net assimilation [32]. LED combinatorial lighting, especially in the absence of natural light, requires further research on the optimization of plant light environment, taking into consideration both biotechnologies applied and specific cultivar demands [1]. Emerging new LED lighting technologies trigger research on the development of optimized light receipts for plant biomass production, target compounds biosynthesis, and other biotechnological applications [33,34,35].

The specific microenvironment conditions in vitro with limited gas exchange, low light, and carbohydrates content in the nutrient medium often promote the production of reactive oxygen species and trigger oxidative stress in plants [36] that can affect plant photosystems. The plants transplanted ex vitro are affected by stressful environments as well, emphasizing underdeveloped and damaged root system, non-functional stomata, and related water stress under low levels of air humidity [37,38].

The studies on the physiological mechanisms of light action on plants, taking into account the new experimental possibilities opened with the use of LEDs, allow us to approach the development of light modes at a fundamentally new level, ensuring the success of plant adaptation in vitro, as well as their ability to overcome the stress of transfer to ex vitro conditions.

There are many reports on the successful LEDs applications to promote in vitro growth and morphogenesis in various plant species due to the possibility to optimize light environment spectral quality [39,40]. Better growth and ex vitro survival rate and biomass yield have been reported under various LED treatments [41,42,43,44,45,46,47].

At the same time, there are few publications on the effects of LED lighting on raspberry, *Rubus idaeus* L., micropropagation in vitro. Red LEDs increased multiplication and rooting of shoots of two raspberry cultivars in comparison to the fluorescent lamps [48], while in another experiment, mixed LED light was less efficient for red raspberry multiplication in comparison to fluorescent lights but still with higher shoot quality [49].

Our objective was to characterize micropropagated raspberry plants acclimation ex vitro in various light environments after transplanting from in vitro conditions. We estimated their photosynthetic and growth adaptation to diverse spectra containing combinations of red, far-red, and blue light (the first series, emphasizing phytochrome effects) and combinations of red, green, and blue light (the second series) to determine how light signals in complex spectra interact to influence growth, photosynthesis, and acclimation.

## 2. Results

Two experiments with different light treatments were carried out. Both experiments were conducted simultaneously, and the plant material from the same stock was used.

### 2.1. Raspberry Plant Responses to Various Red–Far-Red Light Ratios

**Figure 1:**
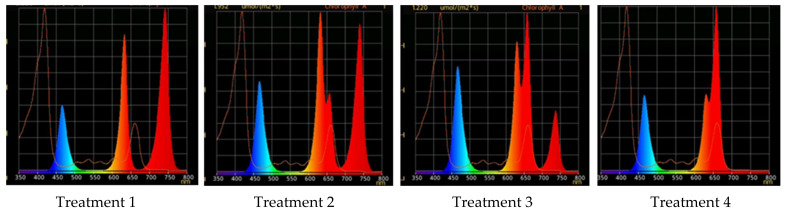
Light treatment spectra, relative portion of red, λ_max_ = 660 nm (R_660_), and far-red, λ_max_ = 739 nm (FR), in their combination: Treatment 1—0% R_660_ + 100% FR; Treatment 2—30% R_660_ + 70% FR; Treatment 3—70% R_660_ + 30% FR; Treatment 4—100% R_660_ + 0% FR.

In this experiment, raspberry plants, *Rubus idaeus* L., cv. Orange Miracle, following in vitro phase, were transplanted in the pots with a peat-based substrate and grown ex vitro at different ratios of red (R)–far-red (FR) light; blue light PPFD was at the same level in all the spectral treatments (Figure 1). Taking into consideration differences in phytochrome *P_r_* and *P_fr_* spectral absorptance [7], we have set different R–FR spectral treatments changing photosynthetic photon flux (PPF) of narrow-band LEDs with irradiance peaking at 730 and 660 nm (light environments precise description is given in Section 4). Additionally, LEDs with λ_max_ = 632 nm were applied to compensate red region photosynthetic photon flux density (PPFD) possible decrease in accordance with experimental set-up; combined R_632_ + R_660_ PPFD had been adjusted to the same level in all treatments.

#### 2.1.1. Growth Responses

**Figure 2:**
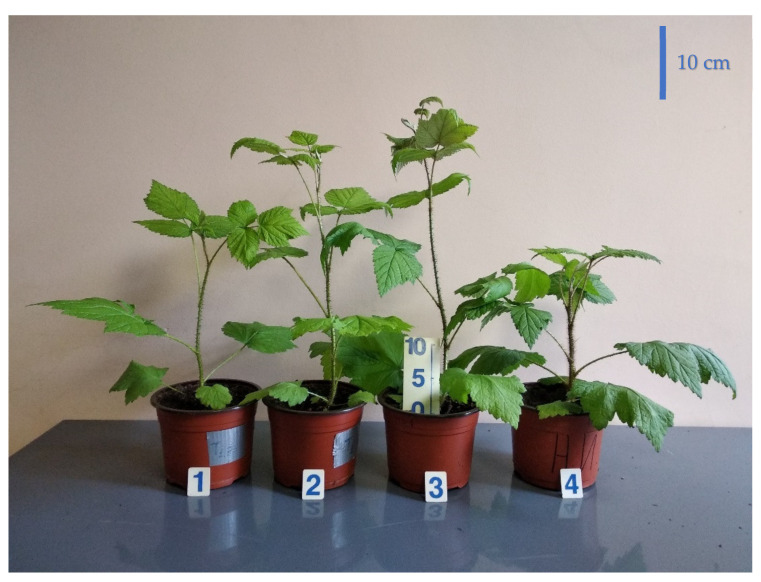
Raspberry plant growth responses to different red (R_660_)–far-red (FR) ratio in the light spectrum. Photo of representative plants, 45 days after transplanting. Treatment 1—0% R_660_ + 100% FR; Treatment 2—30% R_660_ + 70% FR; Treatment 3—70% R_660_ + 30% FR; Treatment 4—100% R_660_ + 0% FR.

**Figure 3:**
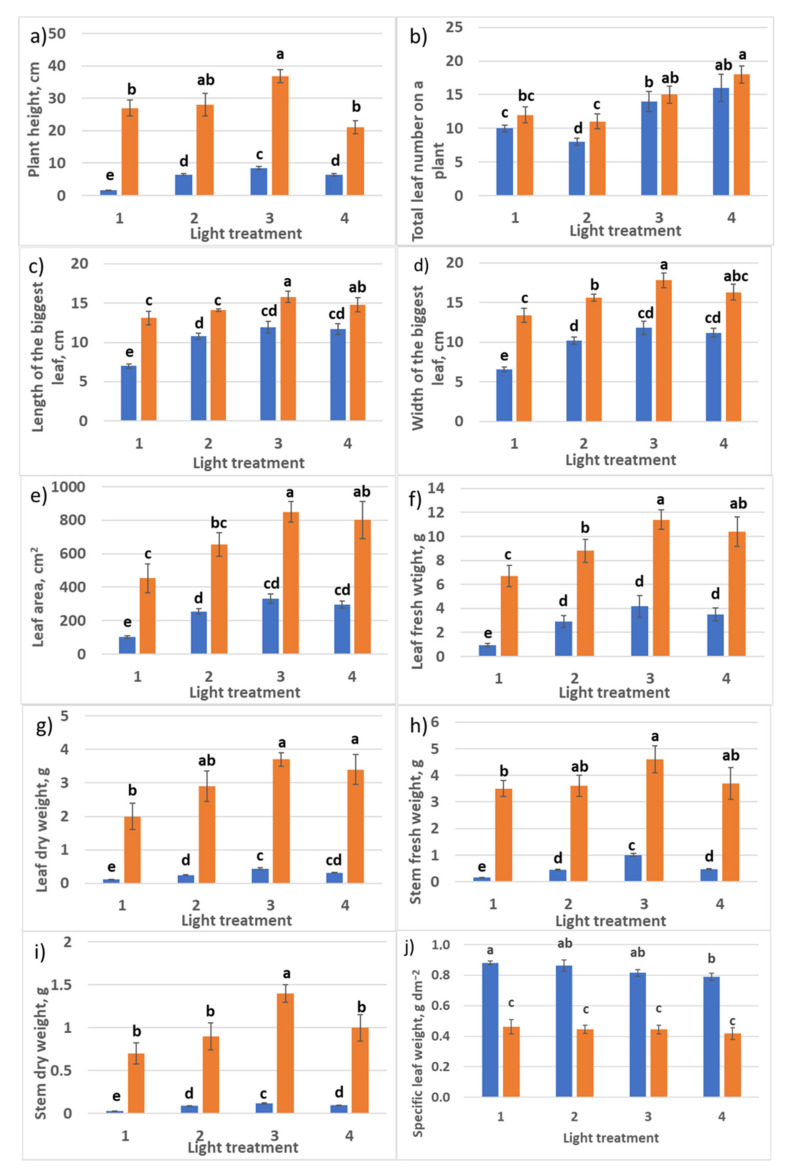
Growth parameters of raspberry plants as affected by different red (R_660_)–far-red (FR)light ratio. Sampling 30 days (blue columns) and 60 days (orange columns) after transplanting. Means ± standard error (SE); means followed by. (**a**) Plant height; (**b**) total leaf number per plant; (**c**) length of the biggest leaf; (**d**) width of the biggest leaf; (**e**) total leaf area; (**f**) total leaf fresh weight; (**g**) total leaf dry weight; (**h**) stem fresh weight; (**i**) stem dry weight; (**j**) specific leaf weight. Means followed by the same letter were not different at *p* ≤ 0.05. Treatment 1—0% R_660_ + 100% FR; Treatment 2—30% R_660_ + 70% FR; Treatment 3—70% R_660_ + 30% FR; Treatment 4—100% R_660_ + 0% FR.

Application of light sources with different ratios of the peak spectral outputs at 660 nm and 730 nm, corresponding to the peak spectral absorptances of phytochrome isoforms *P_r_* and *P_fr_*, respectively, affected raspberry plants acclimation ex vitro. In response to treatment 1 conditions, 20% of plants did not survive after transplanting into a new stressful environment. In the other treatments 2–4, 100% of plants survived. Differences in the ratio of red–far-red light influenced plant growth and morphology (Figure 2 and Figure 3). Light conditions in treatment 1 delayed plant stem elongation during the first month of vegetation (probably due to the general stress responses), but by the end of the second month, the differences between treatments smoothed. New metameres (leaves) formation was also delayed in treatments 1 and 2 with a high portion of FR light. The absence of R_660_ (treatment with 100% FR) inhibited leaf growth significantly by the end of the first month of vegetation (Figure 3c,d); probably, general stress enhanced this response. In treatment 3, activated leaf blade and petiole growth, as well as stem elongation, were observed. Phenotypically, one could connect them with shade-avoidance growth responses, but the R–FR ratio in this treatment was relatively high. Leaf and stem biomass accumulation, as observed earlier, were accelerated by the end of the first month; at the second sampling, these differences were not significant due to increased variation. Plants in treatment 4 produced stem much thicker than in the first three treatments (Figure 2). 

One commonly reported plant morphological response is specific leaf weight (SLW). SLW represents an investment by the plant per unit of leaf area developed, so that plants with the same plant-level net photosynthesis could have very different leaf areas due to differences in SLW and different net photosynthesis rates per unit leaf area. SLW was not affected by various spectral treatments. It is important to note that SLW decreased significantly in all the treatments after the second month of vegetation. This response could be attributed to the plant adaptation to the low irradiation level in the experiment (75 µmol m^−2^ s^−1^). Below, in Section 2.2.1, we return to the analysis of SLW. Root growth was retarded in treatments 3 and especially 4 without FR light. So, shoot–root ratio in biomass accumulation in treatments 1 and 2 with high FR light portion was shifted to roots, and in treatments 3 and 4, to shoots.

#### 2.1.2. CO_2_–H_2_O Exchange

**Figure 4:**
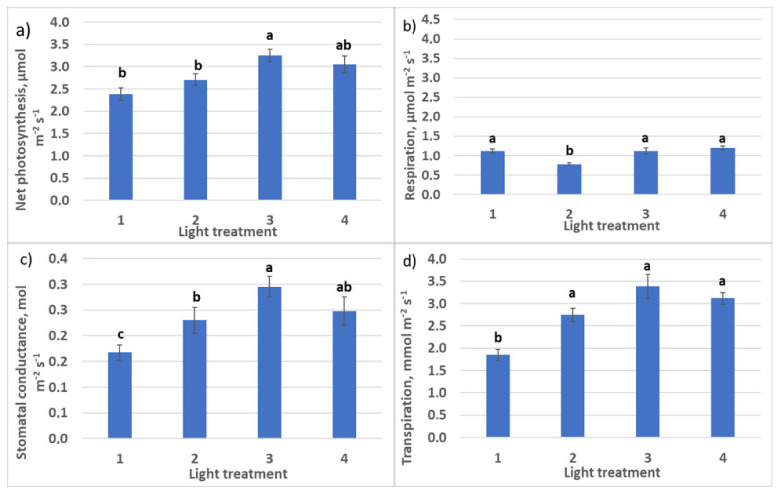
CO_2_–H_2_O leaf exchange in raspberry plants as affected by different red (R_660_)–far-red (FR)light ratio 30 days after transplanting: (**a**) net photosynthesis; (**b**) respiration rate; (**c**) stomatal conductance; (**d**) transpiration rate. Means ± standard error (SE); means followed by the same letter were not different at *p* ≤ 0.05. Treatment 1—0% R_660_ + 100% FR; Treatment 2—30% R_660_ + 70% FR; Treatment 3—70% R_660_ + 30% FR; Treatment 4—100% R_660_ + 0% FR.

Increasing R–FR ratio from light treatment 1 to light treatment 4 resulted in an increase of net photosynthesis rate in plants from 2.4 to 3.1 µmol m^−2^ s^−1^ (Figure 4) and a decrease of respiration–photosynthesis ratio from 0.46 to 0.30–0.40. At the same time, the transpiration rate increase was observed, the most significant in the treatment with 30% FR from R + FR. Assimilation–transpiration ratio (water use efficiency, WUE) was decreasing from regime 1 to regime 4 (1.26, 1.0, 0.85, and 1.0, respectively) due to the increasing transpiration rate.

**Figure 5:**
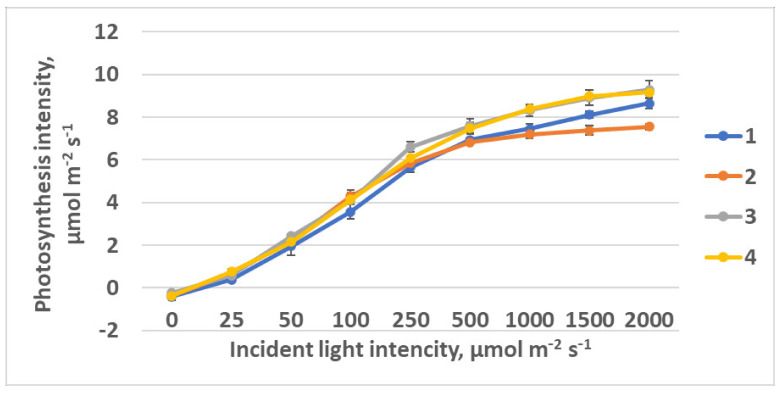
Light response curves of raspberry plants grown under different red (R_660_)–far-red (FR)light ratio. Means ± standard error (SE). Treatment 1—0% R_660_ + 100% FR; Treatment 2—30% R_660_ + 70% FR; Treatment 3—70% R_660_ + 30% FR; Treatment 4—100% R_660_ + 0% FR.

Light curves were approximated using model [50] with the program developed by [51]. Rising of the photosynthesis light response curve plateau was observed in plants as the FR light portion was decreasing from treatment 1 to treatment 4 (Figure 5). Simultaneously, photosynthesis quantum effectiveness was increasing (Table 1). R_640_ could affect the phytochrome system as a very specific light environment. An increase in photosynthesis at saturating light intensity was observed as FR light ratio was decreasing. 

Rising of the photosynthesis light response curve plateau was observed in plants as the FR light portion was decreasing from treatment 1 to treatment 4. Simultaneously, photosynthesis quantum effectiveness was increasing (Table 1). R_640_ could affect the phytochrome system as a very specific light environment. An increase in light intensity at saturation was observed as FR light ratio was decreasing.

#### 2.1.3. Photosynthetic Pigments

**Figure 6:**
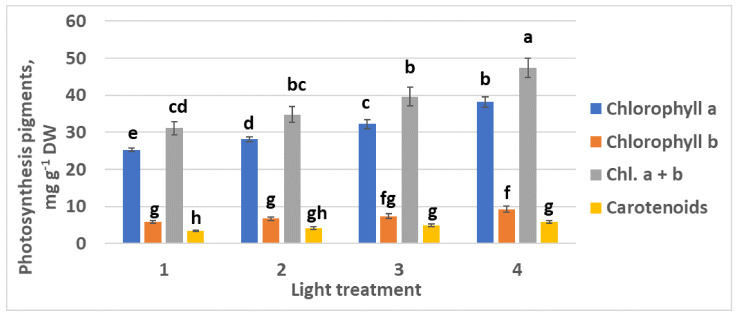
Chlorophylls and carotenoids content in the leaves of raspberry plants grown under various red (R_660_)–far-red (FR) light ratio. Means ± standard error (SE); means followed by the same letter were not different at *p* ≤ 0.05. Treatment 1—0% R_660_ + 100% FR; Treatment 2—30% R_660_ + 70% FR; Treatment 3—70% R_660_ + 30% FR; Treatment 4—100% R_660_ + 0% FR.

Chlorophylls *a* and *b* content increased as the FR ratio in the PPF decreased (Figure 6). Interestingly, relatively high ratio chlorophyll *a*–chlorophyll *b* was observed in all the experimental treatments from 1 to 4: 4.36, 4.19, 4.35, and 4.15, respectively. There was no significant increase in carotenoid content.

#### 2.1.4. Chlorophyll *a* Fluorescence

**Figure 7:**
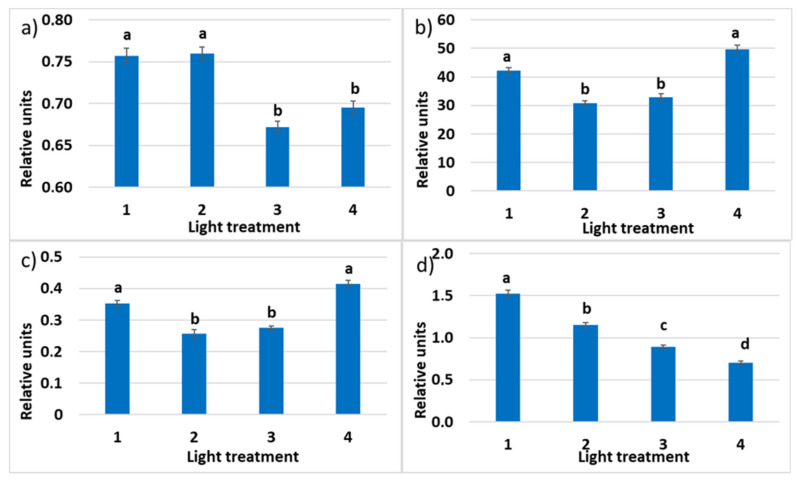
(**a**) Maximum quantum efficiency (Fv/Fm) of photosystem II (PSII); (**b**) photochemical electron transport rate (ETR); (**c**) relative PSII operating efficiency (ΦPSII); (**d**) chlorophyll *a* non-photosynthetic quenching (NPQ) in the leaves of raspberry plants grown under various red (R_660_)–far-red (FR) light ratio. Means ± standard error (SE); means followed by the same letter were not different at *p* ≤ 0.05. Treatment 1—0% R_660_ + 100% FR_730_; Treatment 2—30% R_660_ + 70% FR_730_; Treatment 3—70% R_660_ + 30% FR_730_; Treatment 4—100% R_660_ + 0% FR_730_.

The maximum quantum efficiency of photosystem II (PSII) photochemistry (Fv/Fm) was the highest in treatments 1 and 2 with a high ratio of the FR radiation (Figure 7). It was significantly lower and under 0.83—indicating mild stress [52] —for treatments 3 and 4 lacking FR radiation. (Other authors suggest 0.75 as the bottom border for the healthy unstressed leaves with a normally developed photosynthetic apparatus [53].) It shows that some disorders could occur in the photosynthetic apparatus. To understand how different lighting strategies to influence plant growth, it is important to determine ΦPSII since this partly determines how efficiently they use the absorbed light for photosynthesis and biomass production [54]. Decreased ratio of R_660_ in the spectrum of incident light resulted in the increased quantum yield of photosystem II. The relative operating efficiency of PSII was the highest in the fourth treatment without FR light, decreased in the first treatment, and the lowest in treatments 2–3, combining both R_660_ and FR radiation. A similar mode of response was observed in the changes of the photochemical electron transport (ETR). Chlorophyll *a* non-photosynthetic quenching (NPQ) characterizes absorbed light energy heat dissipation; it was decreasing significantly in response to the decrease in the FR light proportion in the experimental optical radiation spectra from 1 to 4.

### 2.2. Raspberry Plant Responses to Variation in Red, Green, and Blue Light Distribution in the Spectrum of Optical Radiation

**Figure 8:**
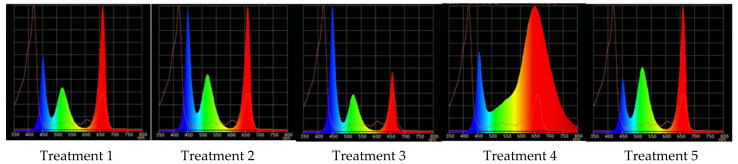
Light treatment spectra, red (R), green (G), and blue (B) light LEDs combinations: Treatment 1–3, 5—“blue” (∆λ_0.5_ = 435 ÷ 458 nm, λ_max_ = 447 nm), “green” (∆λ_0.5_ = 500 ÷ 540 nm, λ_max_ = 518 nm), “red” (∆λ_0.5_ = 645 ÷ 666 nm, λ_max_ = 656 nm); Treatment 4 (control)—“white” (COB, T_color_ = 2500K).

Application of light sources with triple peak spectral outputs at 659 nm, 518 nm, and 450 nm with various ratios of red, green, and blue light, respectively (Figure 8, Table 2), was favorable for growing raspberry plants ex vitro. All the plants, without exceptions, survived after transplanting from in vitro conditions and passed acclimation to the stressful environment with various light spectral conditions successfully.

#### 2.2.1. Growth Responses

**Figure 9:**
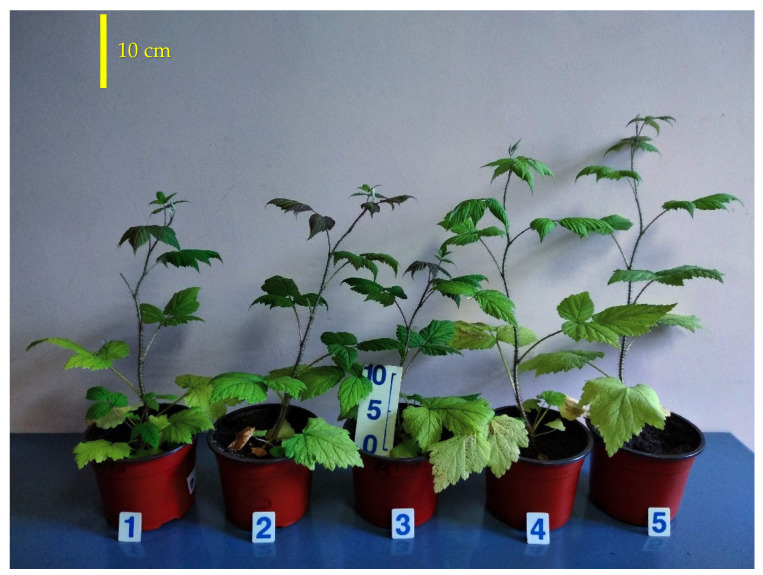
Raspberry plant growth responses to variation in red, green, and blue light distribution in the spectrum of optical radiation. Photo of representative plants, 45 days after transplanting. Treatment 1–3, 5—“blue” (B, ∆λ_0.5_ = 435 ÷ 458 nm, λ_max_ = 447 nm), “green” (G, ∆λ_0.5_ = 500 ÷ 540 nm, λ_max_ = 518 nm), “red” (R, ∆λ_0.5_ = 645 ÷ 666 nm, λ_max_ = 656 nm); Treatment 4 (control)—“white” (chip-on-board, T_color_ = 2500K). Color breakdown: Treatment 1—50% R, 25% G, 25% B; Treatment 2—37% R, 26% G, 37% B; Treatment 3—24% R, 28% G, 48% B; Treatment 4—63% R, 16% G, 21% B; Treatment 5—46% R, 30% G, 23% B.

**Figure 10:**
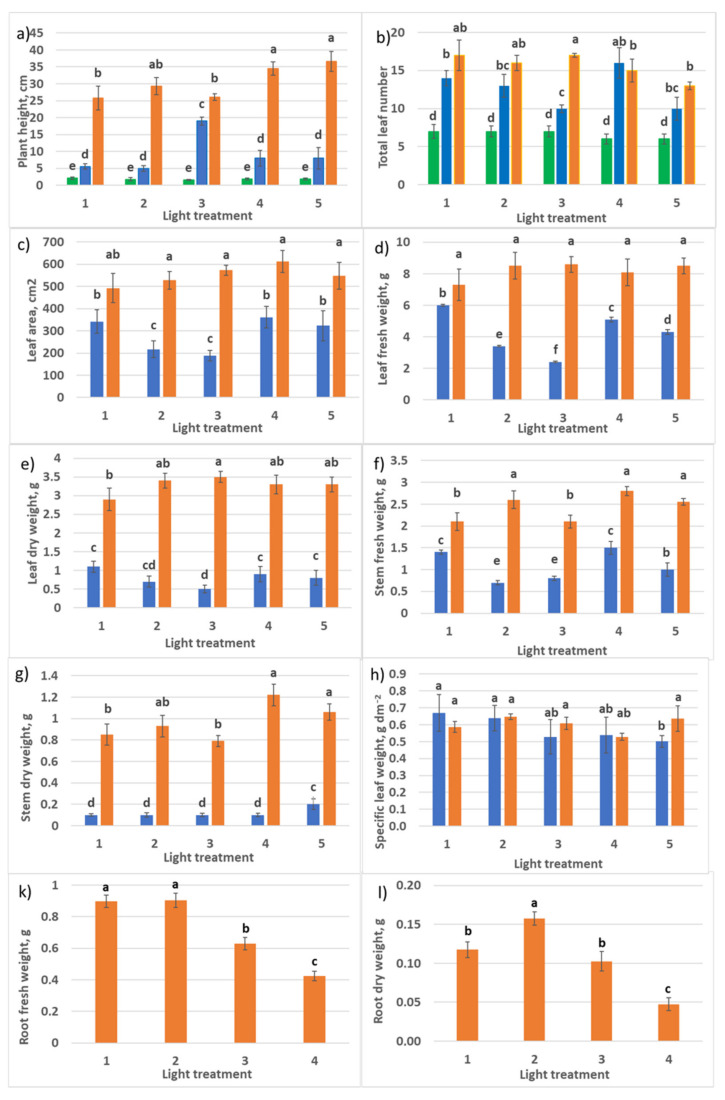
Growth parameters of raspberry plants as affected by different red (R)–green (G)–blue (B) light ratio. Sampling 1 day (green columns), 30 days (blue columns), and 60 days (orange columns) after transplanting. Means ± standard error (SE); means followed by the same letter were not different at *p* ≤ 0.05. (**a**) Plant height; (**b**) total leaf number per plant; (**c**) total leaf area; (**d**) total leaf fresh weight; (**e**) total leaf dry weight; (**f**) stem fresh weight; (**g**) stem dry weight; (**h**) specific leaf weight; (**k**) root fresh weight; (**l**) root dry weight. Treatment 1—50% R, 25% G, 25% B; Treatment 2—37% R, 26% G, 37% B; Treatment 3—24% R, 28% G, 48% B; Treatment 4—63% R, 16% G, 21% B; Treatment 5—46% R, 30% G, 23% B.

Transition to different light regimes influenced plant growth and morphogenesis (Figure 9 and Figure 10).

According to the experimental layout, PPFD in treatments 1–3 consisted almost of the same amount of G light (maximum variation in PPFD level within 12%), and the ratio R–B was changing—2:1, 1:1, and 1:2, respectively. During the first 30 days after transplanting, enhanced stem growth and rapid internode elongation were observed in treatment 3 with an increased portion of B. Since a great part of assimilates was invested in the stem growth, this retarded leaf setting (decreased leaf number) and leaf area development. In other treatments, there were no significant differences between these phenotype parameters. Increased R light portion in treatments 1, 4, and 5 resulted in rapid leaf growth and dry biomass accumulation, and 30 days later (60 days after transplanting), these characters were smoothed in all five treatments. The initial delay in leaf growth in treatments 2 and 3 with increased blue light portion resulted in their slow leaf and stem biomass accumulation. SLW was the same in all the treatments at both samplings. Interestingly, there was no reduction of SLW in older plants, as observed in Section 2.1.1. (PPFD in RGB experiment discussed here was two times higher than in the R–FR experiment.)

#### 2.2.2. CO_2_–H_2_O Exchange

**Figure 11:**
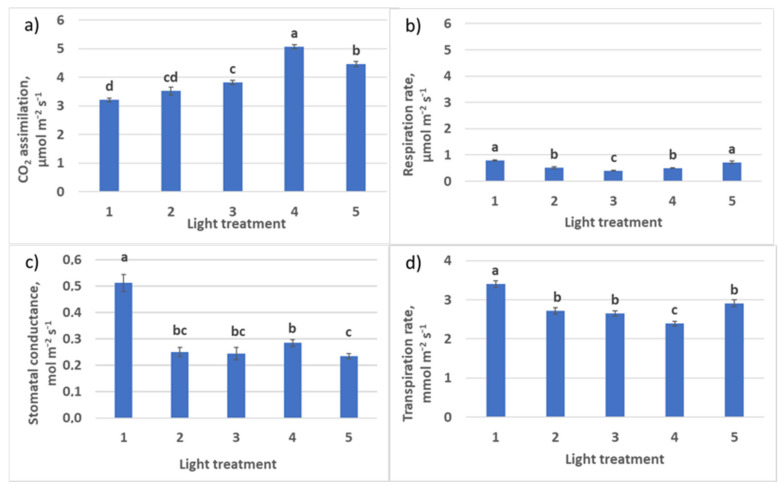
CO_2_ –H_2_O leaf exchange in raspberry plants as affected by different red (R)–green (G)–blue (B) light ratio. (**a**) Net photosynthesis; (**b**) respiration rate; (**c**) stomatal conductance; (**d**) transpiration rate. Means ± standard error (SE); means followed by the same letter were not different at *p* ≤ 0.05. Treatment 1—50% R, 25% G, 25% B; Treatment 2—37% R, 26% G, 37% B; Treatment 3—24% R, 28% G, 48% B; Treatment 4—63% R, 16% G, 21% B; Treatment 5—46% R, 30% G, 23% B.

Decreasing R–B ratio from light treatment 1 to treatment 3 resulted in the increase of net photosynthesis rate in plants (Figure 11) and a decrease of respiration–photosynthesis ratio from 0.25 to 0.11. The transpiration rate also decreased. Calculated assimilation–transpiration ratio (WUE) was increasing from regime 1 to regime 3 (0.94, 1.30, and 1.41, respectively). Stomatal conductance decreased significantly in the treatments with a high blue light portion (R–B = 1:1 and 1:2). In spite of low stomatal conductance in treatments 4 (control) and 5 together with increased green light ratio (treatment 5), there was observed the highest intensity of photosynthesis. Here, it is necessary to stress that there was a large portion of FR light in the photon flux in the reference Treatment 4.

**Figure 12:**
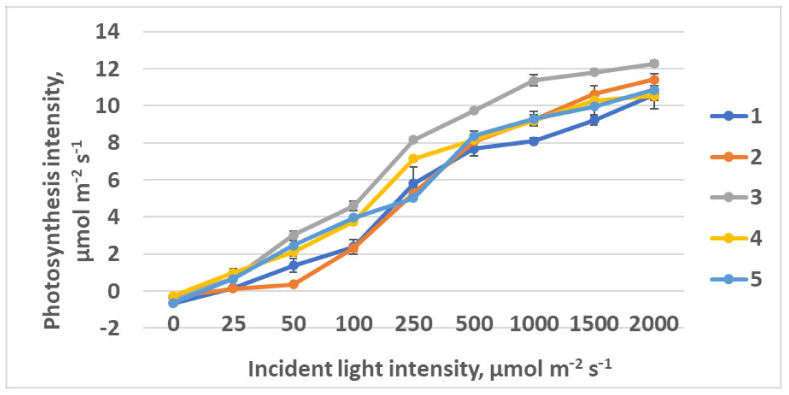
Light response curves of raspberry plants grown under various red (R)–green (G)–blue (B) light ratio. Means ± standard error (SE); means followed by the same letter were not different at *p* ≤ 0.05. Treatment 1—50% R, 25% G, 25% B; Treatment 2—37% R, 26% G, 37% B; Treatment 3—24% R, 28% G, 48% B; Treatment 4—63% R, 16% G, 21% B; Treatment 5—46% R, 30% G, 23% B.

The lowest photosynthesis intensity at the saturating PPFD was observed in treatment 4 (control), with the highest portion of R light in the spectrum (Figure 12, Table 3). Here, low light intensity at light response curve saturation was found, as well. This kind of response is typical for the plants originating from the shaded habitats. The highest photosynthesis at saturating light intensity, 10.2 µmol CO_2_ m^−2^ s^−1^, was observed in treatment 3 with R–B = 1:2. The highest photosynthesis quantum effectiveness was also registered in treatment 3, as well as the lowest light compensation point.

#### 2.2.3. Photosynthetic Pigments

**Figure 13:**
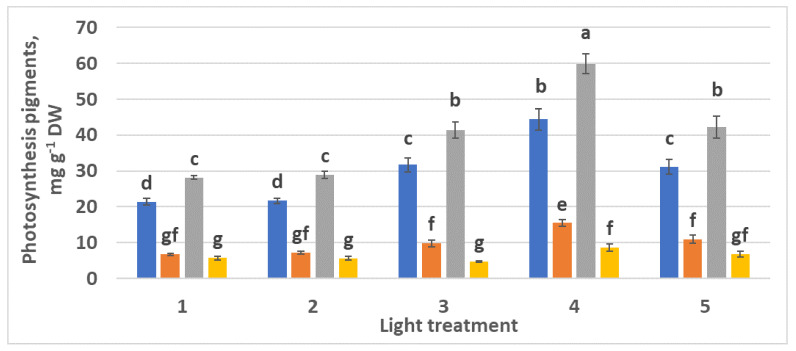
Chlorophylls and carotenoids content in the leaves of raspberry plants grown under various red (R)–green (G)–blue (B) light ratio. Means ± standard error (SE); means followed by the same letter were not different at *p* ≤ 0.05. Treatment 1—50% R, 25% G, 25% B; Treatment 2—37% R, 26% G, 37% B; Treatment 3—24% R, 28% G, 48% B; Treatment 4—63% R, 16% G, 21% B; Treatment 5—46% R, 30% G, 23% B.

The highest chlorophyll *a* and *b* accumulation was observed in the control plants (Figure 13, treatment 4). Additionally, comparing treatments 1 and 5, one can notice a considerable increase in chlorophylls content in response to increased green light portion with the same ratio R–B = 2:1. Chlorophyll *a*–*b* ratios in spectral treatments 1–5 were 3.6:1, 2.8:1, 2.94:1, 2.86:1, and 2.85:1, respectively. These ratios appeared to be more “typical” than those observed in the experiment with R–FR (see Section 2.1.3). The highest content of carotenoids was observed in control plants (treatment 4).

#### 2.2.4. Chlorophyll *a* Fluorescence

**Figure 14:**
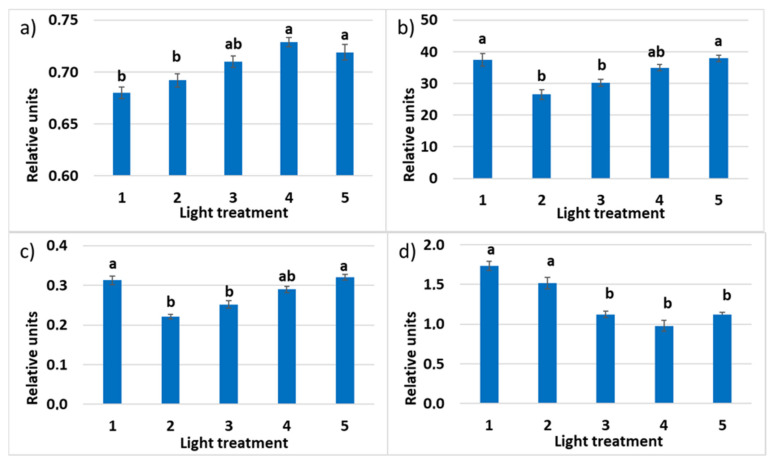
(**a**) Maximum quantum efficiency of PSII (Fv/Fm); (**b**) photochemical electron transport rate (ETR); (**c**) relative PSII operating efficiency (ΦPSII); (**d**) chlorophyll *a* non-photosynthetic quenching (NPQ) in the leaves of raspberry plants grown under various red (R)–green (G)–blue (B) light ratio. Means ± standard error (SE); means followed by the same letter were not different at *p* ≤ 0.05. Treatment 1—50% R, 25% G, 25% B; Treatment 2—37% R, 26% G, 37% B; Treatment 3—24% R, 28% G, 48% B; Treatment 4—63% R, 16% G, 21% B; Treatment 5—46% R, 30% G, 23% B.

The maximum quantum efficiency of PSII photochemistry (Fv/Fm) was comparable in all the spectral treatments (Figure 14). It was under 0.83, indicating mild stress in plants. The increased portion of green light favored the increase of Fv/Fm; however, the difference between treatments 1 and 5 was not significant. The relative operating efficiency of PSII was the highest in treatments 1, 4, and 5 with a high portion of red light in PPFD (50%, 63%, and 46%, respectively), decreased in the third treatment, and was the lowest in treatment 2. A similar mode of response was observed in the changes of the photochemical electron transport (ETR). Chlorophyll *a* non-photosynthetic quenching (NPQ) was decreasing significantly in response to the decrease of the R–B light ratio (treatments 1, 2, and 3, respectively). 

## 3. Discussion

The consequences of plant raising in vitro influence their growth after transplanting ex vitro. First, they affect plant water regime, photosynthetic capacity, and growth. All these physiological functions are interdependent, and limitations in any of them influence the whole physiological machinery increasing various disorders. At the same time, all these functions are light-sensitive, and plants effectively use light signaling to cope with environmental changes. From this viewpoint, the idea to use light manipulations with LEDs for fine-tuning plant physiological processes looks very promising.

The study of the physiological mechanisms of light action on plants, taking into account the new experimental possibilities opened with the use of LEDs, allows us to approach the development of light modes at a fundamentally new level, ensuring the success of plant adaptation in vitro, as well as their ability to overcome the stress of transfer to ex vitro conditions. Plant photosynthesis action spectrum matches with blue and red regions of photosynthetically active radiation in the natural environment [4,5,6]. In artificial lighting, a combination of red and blue light provides increased plant photosynthesis and productivity [22,23,55]. Therefore, these two spectral regions were the basic in our photobiological studies. In the experiments, these spectral regions were supplemented with physiologically active far-red or green light.

### 3.1. Raspberry Plant Responses to Various Red–Far-Red Light Ratios

Disrupted water balance control due to the low stomata functional activity after the in vitro environment [37] can seriously affect plant growth and even survival ex vitro. A new light environment could become another stressor. Artificial light sources based on red, green, and blue LEDs are completely deficient in FR wavelength as compared to high-pressure sodium (HPS) lamps or natural irradiance. This results in an increased PSII excitation. The photosystems stoichiometry could be improved by adding FR light which is a “PSI” light [56]. There are data that FR enhances PAR (400–700 nm) quantum yield in various species [57,58]. It is important to note that plant responses to various stressors involve a phytochrome regulatory system; phytochrome effects on photosynthesis are fortified under stress conditions [59].

Increased plant growth is usually associated with a higher fraction of far-red light [60,61]. According to the Emerson effect, both the red and far-red bands are significant contributors to the photosynthetic process for plants [62]. In recent studies, special treatments indicated that the absorbed far-red photons were equally efficient for photosynthesis when acting synergistically with the 400–700 nm photons [58]. Additionally, a reduced ratio of red–far-red light due to the phytochromes involvement triggers shade-avoidance responses in plants [32,63,64,65], which can improve plant photosynthetic capacity [66]. Shade avoidance enables plants to anticipate future competition with the neighbors for light by reducing reliance on resources for branching and capitalizing more on stem elongation and leaf area development in the canopy upper level [67]. The increased portion of far-red radiation (FR, 700–800 nm) relative to photosynthetically active radiation (PAR, 400–700 nm) may induce stem elongation and leaf expansion, which can optimize light interception, and FR might even be photosynthetically active in combination with PAR [65]. The increased growth rate in response to far-red also affects plant source–sink relations: high assimilate demand results in increased photosynthetic activity. Thus, additional far-red LED irradiance usually increases plant size and total yield [68,69]. 

Relatively low R–FR ratio in our experiment can be perceived by plants as a signal triggering shade-avoidance response, and in spite of the minimum direct contribution of FR to photosynthesis, its addition can enhance photoassimilation [57]. Nevertheless, in our studies, photosynthetic apparatus, primary photosynthetic responses were more efficient in treatment 4 without FR light. Still, it was the level of the leaf, and within a whole plant, integral effects could be different. The effect of decreasing the R–FR ratio on leaf area is species specific [8]. In our experiment, far-red light mediated decreased leaf area, which is different from the responses in other plants [70].

Interestingly, a high portion of FR in treatments 1 and 2 resulted in a rapid growth of roots which is favorable for the production of planting material. Above-ground plant habit was more attractive in treatment 4, excluding FR radiation. 

Absorbed photons energy can be used in the light reactions of photosynthesis to drive photochemistry (electron transport), while some amount of light energy is dissipated as heat (NPQ, non-photochemical quenching) or re-emitted as fluorescence. The ratio of the photon excitation energy stream division between the photochemical reactions on the one hand and NPQ and chlorophyll fluorescence, on the other hand, determines the effectiveness of photosynthetic apparatus (primary photosynthesis reactions) [71].

The maximum quantum efficiency of PSII photochemistry (Fv–Fm) was relatively low in Treatments 3 and 4. It indicates how effectively PSII uses absorbed light energy to reduce the primary quinone acceptor of PSII (QA); in practice, this indicator can be used to assess stress in plants [52,53]. Values below 0.75–0.83 indicate stress and a reduced maximum photosynthetic capacity; however, photosynthesis may not be reduced under ambient conditions as the quantum yield of PSII (Φ_PSII_) is usually lower than Fv–Fm [8]. A low Fv–Fm is one of the symptoms of red light syndrome [12,13,56]. Low Fv–Fm rates in Treatments 3 and 4 indicated a reduced maximum photosynthetic efficiency with values suggesting mild stress.

Higher chlorophyll content in leaf blades in the treatments with decreased FR PPFD provides better conditions for the increased light absorption, as it was demonstrated earlier for woody plants [72]. The lowest NPQ was also observed in the treatment without FR radiation. 

Thus, in our experiments, higher effectiveness of photosynthetic apparatus (primary light reactions) was observed in response to the irradiance with a decreased ratio of FR light. However, plants in these treatments had a higher transpiration rate that can be regarded as a disadvantage at the initial phase of adaptation.

### 3.2. Raspberry Plant Responses to Variation in Red, Green, and Blue Light in the Spectrum of Optical Radiation

Speaking about light environment optimization for plant growing, one must keep in mind that the effects of the blue and red lights are not equal: red light is perceived in addition to photosynthetic apparatus by phytochromes only, whereas blue light is absorbed by both phytochromes and blue light receptors (cryptochromes and phototropins). In this respect, blue light exciting a larger set of photoreceptors is functionally more versatile [73]. Of course, photomorphogenetic responses are characterized by a very low fluence rate in comparison with photosynthesis.

In our experiment, blue light significantly enhanced photosynthetic capacity relative to treatments lacking blue light, which is consistent with other findings, e.g., analyses of Rubisco activity and potential rate of photosynthetic electron transport [8]. In leafy crops, additivity in combined effects of R and B on plant productivity was observed in lettuce, but not in sweet basil plants [16].

An increase in A_max_ with increasing blue light percentage could be associated with an increase in specific leaf weight, chlorophyll content per area, and stomatal conductance, as it was observed before [11]. However, A_max_ growth in our case (in treatments with blue light percentage increase from 25% to 48%) was not so significant as in [11], where binary red-blue spectra were applied.

A broader spectrum can result in higher CO_2_ fixation than targeted red–blue light treatment like it was shown for tomato and poinsettia but not in cucumber at ambient CO_2_ concentrations [74]. 

The leaf higher photosynthetic capacity was connected with an increased portion of blue light in the spectrum. A higher blue–red light ratio in our experiments resulted in plant photomorphogenetic responses, e.g., they produced leaves with properties typical for the plants grown at high PPFD levels. This could be advantageous for the production of “high-light acclimated” plants at the nursery with a poor irradiance, as it was considered earlier [56].

A higher level of blue light in LED treatments increased Fv–Fm; there are data that blue light enhances PSII photochemistry relative to red light [8]. Chlorophyll *a* non-photosynthetic quenching (NPQ) characterizes absorbed light energy heat dissipation. It has decreased significantly only in response to red light proportion fall from 50% to 37% (treatments 1 and 2, respectively). Both high Φ_PSII_ and high chlorophyll content may increase the ETR, CO_2_ assimilation rate, and potentially the growth rate of plants [54], as it was also observed in treatment 5 in our experiment. 

The green spectral region portion in the artificial PPFD provides better light penetration into the canopy and optimize plant growth adaptations in the coenosis [75,76]. Additionally, green light improves photosynthetic capacity, e.g., relative to monochromatic red light [8]. It was shown earlier that G light stimulates stem elongation and stomatal closure, antagonizing the typical blue-light mediated growth inhibition and stomatal opening [29,30,77]. The effects of green light tend to reverse the processes established by red and/or blue light. In this way, a green light may be functioning in a manner similar to far-red light, informing the plant of photosynthetically unfavorable conditions and triggering adaptative responses [78].

Our studies have shown that in triple spectral combinations increased portion of green light or blue light enhanced red light effects on the net leaf photosynthesis (Figure 3). Red and green light enhancement can be explained, at least partly, by the ‘Emerson effect’; according to it, photosynthesis activity from combined spectra can be greater than the sum of its parts due to excitation energy distribution between photosystem I and photosystem II [62,79,80]. 

High-light crop species could respond to the fraction of photons in the green region with either shade tolerance (leaf expansion) or shade avoidance (stem elongation). [81]. In addition, blue photons usually decrease leaf area and dry mass (see Figure 10c–f). Therefore, it is suggested to include a low fraction of blue photons in a light source [81].

Plant responses discussed above are concerning biomass production first. However, depending on the object and technology applied, other goals could be important. In micropropagation, these are stress-resistance and ex vitro survival, as it was mentioned in this article title. Thus, calculated assimilation/transpiration ratio (WUE) was increasing from regime 1 to regime 3 (0.94, 1.30, and 1.41, respectively). Stomatal conductance decreased significantly in the treatments with high blue light portion (R–B = 1:1 and 1:2). If we compare stomatal conductance, net photosynthesis, and respiration–photosynthesis ratio (0.25 and 0.16, respectively) in treatments 1 and 5 (with the same ratio R–B = 2:1, but with an increased portion of green light in the last case), the favorable effect of green light on the assimilation also becomes evident. WUE in treatment 5 was 1.55 (65% higher than in treatment 1). 

## 4. Materials and Methods

The experimental work consisted of 2 experiments with different experimental layouts conducted simultaneously.

### 4.1. Plant Material

Raspberry, *Rubus idaeus* L., plants of the evergreen cultivar Orange miracle were used in our studies. Plants produce vigorous shrubs and are characterized with a high shoot producing capacity. Shoots are covered with thorns. Berries of medium size with orange color and good taste and aroma. Medium draft resistance.

### 4.2. Plant Growing In vitro

Microplants of raspberry Orange miracle grown in vitro were used for planting ex vitro and further research. Aseptically cultured microplants were used for cutting preparation. We used a node with two axillary buds as an ex-plant.

During the micropropagation stage, microplants were clonally propagated by cuttings. To induce the formation of adventitious buds and axillary shoots, ex-plants were cultured on Quoirin and Lepoivre medium (QL), containing 6-benzylaminopurine (6-BAP) and indole-3-acetic acid (IAA) at concentrations of 1.0 and 0.5 mg L^−1^, respectively. During the rooting stage, we used QL medium with half salt concentration, containing 1.0 mg L^−1^ of indole-3-butyric acid (IBA). The pH level of all nutrient media was adjusted to 5.9–6.2.

The sterility of the medium was obtained by autoclaving at 120 °C, 1.3 atm. for 25 minutes. Procedures of cutting preparation were performed in a sterile laminar box. The tools were fired in the burner’s flame immediately before the operation. Ex-plant disinfection was not performed due to the source material asepsis. 

Microplants were cultured in a light room with the temperature of 22–24 °C; lighting was provided with OSRAM L36/25 fluorescent white lamps with at photosynthetic photon flux density 150–180 µmol m^−2^ s^−1^, photoperiod 16 h.

### 4.3. Plant Growing Ex vitro

When microplants formed a well-developed root system (*ca*. 15 days), they were used for further ex vitro adaptation in growth chambers (Urbangrower 150, PR China) with various light treatments according to experiments’ layout. 

Plants were transplanted into the commercial neutralized peat-based substrate “Agrobalt-C” (Pindstrup, Pskov region, Russia) with pH 6.0-6.5 and complete macro- and micronutrient supply including 150 mg L^−1^ [NH_4_^+^ andNO_3_^−^], 270 mg L^−1^ P_2_O_5_, and 300 mg L^−1^ K_2_O. Plants were grown in 2 L vegetational vessels. Substate humidity was maintained at 70% of full water capacity, watering on the scales.

### 4.4. Different Light Conditions Experimental Design

Plant chambers were illuminated with lamps consisting of various light-emitting diode (LED) bars specifically designed to provide a custom spectrum in each chamber. Fixtures consisted of light modules with tunable light-emitting diodes varying in the wavelength and spectral composition of the emitted light over wide ranges. 

In the experiment on the effects of R–FR light ratio, four types of high-performance narrow-band 3 w LEDs (Estar, Cidly, PR China) were used: short-wave red (**∆**λ_0.5_ = 623 ÷ 641 nm, λ_max_= 632 nm), long-wave red (∆λ_0.5_ = 646 ÷ 674 nm, λ_max_= 660 nm), far-red (∆λ_0.5_ = 727 ÷ 751 nm, λ_max_= 739 nm), and blue (∆λ_0.5_ = 452 ÷ 477 nm, λ_max_= 465 nm). In all the light treatments, photosynthetic photon flux density (PPFD) 75 µmol m^−2^ s^−1^ was maintained, photoperiod 18 h. Spectra of the resulting lamp systems were measured with a spectrometer UPRtek PG100N (Taiwan). To measure the PPFD in the PAR region, an LI-191R quantum sensor with an LI-250A data logger (Li-Cor, NE, USA) was used. 

In the experiment on the R–G–B light ratio effects, three types of tunable LEDs (Cree, USA) were used: red (∆λ_0.5_ = 647 ÷ 671 nm, λ_max_= 659 nm), green (∆λ_0.5_ = 500 ÷ 540 nm, λ_max_= 518 nm), and blue (∆λ_0.5_ = 438 ÷ 462 nm, λ_max_= 450 nm). Warm “white” COB with T_color_ = 2500K (Citizen, Japan), also containing FR in the spectrum, was used as a reference treatment in this series (Figure 8).

### 4.5. Plant Growth Analyses

Four plants per each treatment were destructively harvested twice: 30 and 60 days after transplanting. The number of leaves (>1 cm) per plant was counted and total leaf area was measured using a leaf area meter LI-3000A (Li-Cor, NE, USA). Shoot fresh weight was measured using an electronic balance. Subsequently, shoots were oven-dried to a constant weight at 70 °C for dry weight determination. Specific leaf weight (SLW) was calculated by dividing leaf weight by leaf area (dry weight per unit leaf area).

### 4.6. CO_2_–H_2_O Leaf Exchange Determination 

Plant leaf photosynthetic rate and transpiration analyses were carried out using a LI-6400XT Portable Photosynthesis System (Li-Cor, NE, USA). During the measurements, CO_2_ concentration was maintained at 400 ± 12.0 µmol mol^−1^, air temperature 21–23 ° C, air humidity 60 ± 4.0%. Light curves were approximated using model [50] with the program developed by [51].

### 4.7. Photosynthetic Pigments Determination

The quantitative analysis of pigments (chlorophylls and carotenoids) included their extraction from the plant tissues using acetone, separation of the mixture into individual components, and spectrophotometry. The concentrations of pigments in 100% acetone leaf tissue extracts were calculated according to Holm–Wettstein as follows: C_Chla_ = 9.784 × D_662_ − 0.990 × D_644_; C_Chlb_ = 21.426 × D_644_ − 4.650 × D_662_; C_Chla_+_Chlb_ = 5.134 × D_662_ + 20.436 × D_644_; C_car_ = 4.695 × D_440.5_ − 0.268 × C_Chla_+_Chlb._ Here, C_Chla_, C_Chlb_, and C_car_ are the concentrations of chlorophylls *a* and *b* and carotenoids in acetone. D_λ_ is the optical density of the extract at the corresponding wavelength λ. Pigment content was calculated per dry weight.

### 4.8. Chlorophyll a Fluorescence Determination

Chlorophyll *a* fluorescence in PSII was measured using Junior-PAM fluorimeter (Heinz Walz, Germany). Minimum (Fo) and maximum (Fm) fluorescence rates were determined after 15 min leaf exposition in darkness. Maximum quantum efficiency of PSII Fv–Fm = (Fm–Fo)/Fm was calculated after [82]. Relative PSII operating efficiency (ΦPSII) of the light-adapted leaves was calculated as ΦPSII = (F′m–Ft)/F′m. Chlorophyll a non-photosynthetic quenching NPQ = (Fm–F′m)/F′m. photochemical electron transport rate ETR = (Φ_PSII_)·PPFD·0.5. Fluorescence parameters were determined in 4–6 biological replicates. 

### 4.9. Statistical Analysis of Experimental Data

For each light treatment, four replications were tested during plant phenotyping. Statistical analysis of physiological parameters was performed using analysis of variance (ANOVA) with MS Excel software and AGROS software (version 2.11, Russia). In the graphs and tables, means ± standard error (SE) are presented; means followed by the same letter were not different at *p* ≤ 0.05.

## 5. Conclusions

Plant acclimation and photosynthetic improvements in response to added far-red and green light wavelengths to the main red–blue spectrum have been studied along with the changing red–blue light ratio.

Water stress and light stress (transition to a new light environment, abnormal spectral composition) follow in vitro plants transition ex vitro. Acclimation to different growth-light spectra assumes photosystem excitation balance maintenance and plant water status stabilization due to the stomata apparatus improvement and other morphological and functional adaptations. 

Plants can tune their stoichiometry to retain high photosynthetic quantum yield in the artificial non-typical light environment [83]. They have evolved a variety of mechanisms to cope with the changes in light spectral quality and intensity. Thus, light-harvesting complex II (LHC-II) can be transferred from PSII to PSI to help balance excitation energy between the two systems to improve electron transport efficiency [8,84] and adjust the stoichiometry of photosystem I (PSI) and photosystem II (PSII) in response to light quality to improve photosynthetic efficiency as well as their pigment composition for the efficient light absorption [8,85,86].

Sharp plant responses and significant differences between the light treatments in our experiments were observed during the first period after transplanting, at the critical phase of acclimation. It is clear that at this stage, increasing the fractions of green and blue photons in photosynthetic photon flux could benefit plant survival. After this water-saving treatment, tuning of growth and photosynthesis by manipulations with the R–FR ratio and R–B ratio becomes more critical. Thus, in development, we suggest a dynamic (changing in time) light regime combining the advantages of distinct spectra studied above at the critical periods of their action during the initial plant growth. Or course, this dynamic light environment optimization requires further research. There are data on the LED spectra effects on the production of the reactive oxygen species (ROS) in the electron transport system and activation of various plant defense mechanisms [87]. Studies with raspberry plants grown in vitro with quasimonochromatic light, as mentioned before, also demonstrated spectral variation in the LED effects both on the ROS accumulation and scavenger activity [47]. In our future research, we are in a position to add determination of free radical production and scavenging mechanisms activity to justify ex vitro plant light and water stress acclimation evidence along with the photosynthesis and fluorescence parameters.

## Figures and Tables

**Table 1:** Light response curve parameters in raspberry plants grown under different red (R_660_)–far-red (FR) light ratio. Means ± standard error (SE); means followed by the same letter were not different at *p* ≤ 0.05.

Parameters	Light Treatment			
	**1**	**2**	**3**	**4**
R_660_	0%	30%	70%	100%
FR	100%	70%	30%	0%
Photosynthesis at saturating light intensity, µmol CO_2_ m^−2^ s^−1^	9.0 ± 0.07 a	8.0 ± 0.21 b	9.28 ± 0.77 abc	10.2 ± 0.28 c
Dark respiration, µmol CO_2_ m^−2^ s^−1^	0.40 ± 0.01 a	0.48 ± 0.01 b	0.42 ± 0.08 a	0.49 ± 0.02
Photosynthesis quantum yield, µmol CO_2_ m^−2^ s^−1^ µmol m^−2^ s^−1^	0.039 ± 0.013 a	0.069 ± 0.12 b	0.055 ± 0.018 ab	0.066 ± 0.011 b
Light compensation point, µmol m^−2^ s^−1^	10.2 ± 1.0 a	7.0 ± 0.4 b	7.8 ± 0.5 b	7.4 ± 0.6 b
Light intensity at saturation, µmol m^−2^ s^−1^	238 ± 21a	124 ± 12 b	178 ± 19 ac	163 ± 13 c

**Table 2:** Color breakdown for light treatment spectra as a percentage of total photosynthetic photon flux density (PPFD).

Treatment	Red (600–700 nm)	Green (500–600 nm)	Blue (400–500 nm)
1	50	25	25
2	37	26	37
3	24	28	48
4	63	16	21
5	46	30	23

**Table 3:** Light response curve parameters in raspberry plants grown under various red (R)–green (G)–blue (B) light ratios. Standard error (SE); means followed by the same letter were not different at *p* ≤ 0.05.

Parameters	Light Treatment				
	**1**	**2**	**3**	**4**	**5**
R	50%	37%	24%	63%	46%
G	25%	26%	28%	16%	30%
B	25%	37%	48%	21%	23%
Photosynthesis at saturating light intensity, µmol CO_2_ m^−2^ s^−1^	9.3 ± 0.8 b	9.4 ± 0.5 b	10.2 ± 0.3 b	5.4 ± 0.6 a	8.4 ± 0.6 b
Dark respiration, µmol CO_2_ m^−2^ s^−1^	0.54 ± 0.052 a	0.50 ± 0.055 a	0.49 ± 0.01 a	0.47 ± 0.046 a	0.44 ± 0.045 a
Photosynthesis quantum yield, µmol CO_2_ m^−2^ s^−1^/ µmol m^−2^ s^−1^	0.047 ± 0.015 a	0.054 ± 0.018 a	0.066 ± 0.012 b	0.051 ± 0.015 a	0.052 ± 0.015 a
Light compensation point, µmol m^−2^ s^−1^	11.6 ± 0.4 c	9.2 ± 0.5 a	7.4 ± 0.3 b	9.3 ± 0.3 a	8.5 ± 0.3 a
Light intensity at saturation, µmol m^−2^ s^−1^	209 ± 17 b	184 ± 15 b	163 ± 15 b	116 ± 12 a	170 ± 14 b

## Data Availability

Data sharing is not applicable to this article.
